# Autonomic failure in Parkinson’s disease is associated with striatal dopamine deficiencies

**DOI:** 10.1007/s00415-020-09785-5

**Published:** 2020-03-12

**Authors:** Dagmar N. van Deursen, Odile A. van den Heuvel, Jan Booij, Henk W. Berendse, Chris Vriend

**Affiliations:** 1 Anatomy and Neurosciences, Amsterdam Neuroscience, Amsterdam UMC, Vrije Universiteit Amsterdam, De Boelelaan 1117, Amsterdam, The Netherlands; 2 Department of Psychiatry, Amsterdam Neuroscience, Amsterdam UMC, Vrije Universiteit Amsterdam, De Boelelaan 1117, Amsterdam, The Netherlands; 3 Department of Radiology and Nuclear Medicine, Amsterdam Neuroscience, Amsterdam UMC, Academic Medical Center, Meibergdreef 9, Amsterdam, The Netherlands; 4 Department of Neurology, Amsterdam Neuroscience, Amsterdam UMC, Vrije Universiteit Amsterdam, De Boelelaan 1117, Amsterdam, The Netherlands

**Keywords:** Dopamine, Serotonin, Autonomic dysfunction, Parkinson, [^123^I]FP-CIT SPECT

## Abstract

Autonomic dysfunction is a common non-motor symptom in Parkinson’s disease (PD). Dopamine and serotonin are known to play a role in autonomic regulation, and, therefore, PD-related degeneration of serotonergic and dopaminergic neurons in these regions may be associated with autonomic dysfunction. We sought to clarify the association between extrastriatal serotonergic and striatal dopaminergic degeneration and the severity of autonomic symptoms, including gastrointestinal, pupillomotor, thermoregulatory, cardiovascular, and urinary dysfunction. We performed hierarchical multiple regression analyses to determine the relationships between (extra)striatal serotonergic and dopaminergic degeneration and autonomic dysfunction in 310 patients with PD. We used [^123^I]FP-CIT SPECT binding to presynaptic serotonin (SERT) and dopamine (DAT) transporters as a measure of the integrity of these neurotransmitter systems, and the SCOPA-AUT (Scales for Outcomes in Parkinson’s Disease—Autonomic) questionnaire to evaluate the perceived severity of autonomic dysfunction. Motor symptom severity, medication status, and sex were added to the model as covariates. Additional analyses were also performed using five subdomains of the SCOPA-AUT: cardiovascular, gastrointestinal, urinary, thermoregulatory, and pupillomotor symptoms. We found that autonomic symptoms were most significantly related to lower [^123^I]FP-CIT binding ratios in the right caudate nucleus and were mainly driven by gastrointestinal and cardiovascular dysfunction. These results provide a first look into the modest role of dopaminergic projections towards the caudate nucleus in the pathophysiology of autonomic dysfunction in PD, but the underlying mechanism warrants further investigation.

**Electronic supplementary material:**

The online version of this article (10.1007/s00415-020-09785-5) contains supplementary material, which is available to authorized users.

## Introduction

Besides the characteristic motor symptoms, Parkinson’s disease (PD) patients also frequently suffer from a variety of non-motor complaints including cognitive decline, neuropsychiatric problems, and autonomic dysfunction [1]. Autonomic dysfunction is often one of the earliest manifestations of PD. Symptoms of autonomic failure can be cardiovascular, gastrointestinal, urogenital, thermoregulatory, pupillary, respiratory, sexual, or sleep related [2]. The symptoms typically worsen with progression of the disease, and the use of dopamine replacement therapy may also contribute [3]. Examples of symptoms include dysmotility of the gastrointestinal tract, abnormal sweating patterns, detrusor overactivity, and orthostatic hypotension [4–6].

Serotonin (5-hydroxytryptamine; 5-HT) has an important role in the regulation of the autonomic nervous system (ANS). Serotonergic neurons originating from the nucleus raphe obscurus and raphe pallidus have projections to autonomic nuclei in the medulla and spinal cord. These serotonergic neurons also receive input from brain areas important for autonomic regulation in the brainstem, forebrain, and hypothalamus [7, 8]. Serotonin is also involved in numerous descending myenteric interneurons that innervate the enteric nervous system [9]. Increasing or decreasing serotonin signalling affects heat or cold tolerance [10, 11], peristalsis [9], urine storage [12, 13], and blood pressure [14]. In rodents, stimulating serotonergic neurons in the hypothalamus alters energy metabolism and cardiovascular functioning [15, 16]. Dopamine has also been found to play a role in the ANS [17]. Cholinergic myenteric neurons mediate the vagal excitatory effects, especially in the stomach and oesophagus [18]. Some myenteric neurons are dopaminergic, and dopamine affects gastrointestinal motility via presynaptic D2 receptors by inhibiting acetylcholine release [17]. Striatal D2 receptors are also involved in the salivary response [19], the micturition reflex and detrusor activity [20], and central blood pressure and heart rate regulation [21].

Nuclear imaging is routinely used to visualize and measure the integrity of these neurotransmitter systems, using radiotracers that bind to the serotonin (SERT) and dopamine (DAT) transporters in the presynaptic terminal of serotonergic and dopaminergic neurons, respectively. Single-photon emission computed tomography (SPECT) scans using a radiotracer with high affinity for the DAT can be used to detect a loss of nigrostriatal cells in early PD cases, even in the premotor phase [22]. A commonly used DAT tracer is [^123^I]*N*-ω-fluoropropyl-2β-carbomethoxy-3β-(4-iodophenyl)nortropane, or [^123^I]FP-CIT (or [^123^I]ioflupane). This tracer is predominantly useful for assessing dopamine integrity in the striatum, but also has a modest affinity for the SERT located on the presynaptic membrane of serotonergic neurons such as within the (hypo)thalamus and midbrain [23].

Nuclear imaging has previously been used to show serotonergic as well as dopaminergic loss in PD [24, 25]. Nevertheless, to our knowledge, no study has yet investigated the association between the integrity of the serotonergic system and autonomic dysfunction in PD using nuclear imaging. Recent studies have shown relationships between constipation [26] and urinary symptoms [27] and reduced striatal DAT availability, but more research is needed to confirm these findings. In the current study, [^123^I]FP-CIT SPECT imaging was used to investigate the relationship between extrastriatal SERT availability, as well as striatal DAT availability, and the severity of autonomic symptoms in PD. The aim was to provide insight into the pathophysiology of autonomic dysfunction in PD, and evaluate the involvement of these two neurotransmitter systems. Based on previous studies, we hypothesized that lower SERT binding in the hypothalamus and other extrastriatal areas would be associated with increased autonomic symptoms. Likewise, we hypothesized that lower striatal DAT binding would be associated with increased autonomic symptoms.

## Methods

### Participants

There were three inclusion criteria for this study: patients must (1) have been diagnosed with idiopathic PD; (2) have an available [^123^I]FP-CIT SPECT; (3) have provided written informed consent in accordance with the declaration of Helsinki to use their medical information obtained during routine clinical care in the outpatient clinic for research purposes. Imaging data and clinical data were obtained through the outpatient clinic for movement disorders of the department of Neurology, Amsterdam UMC, location VUmc, The Netherlands in the period from 2008 to 2018.

**Fig. 1:**
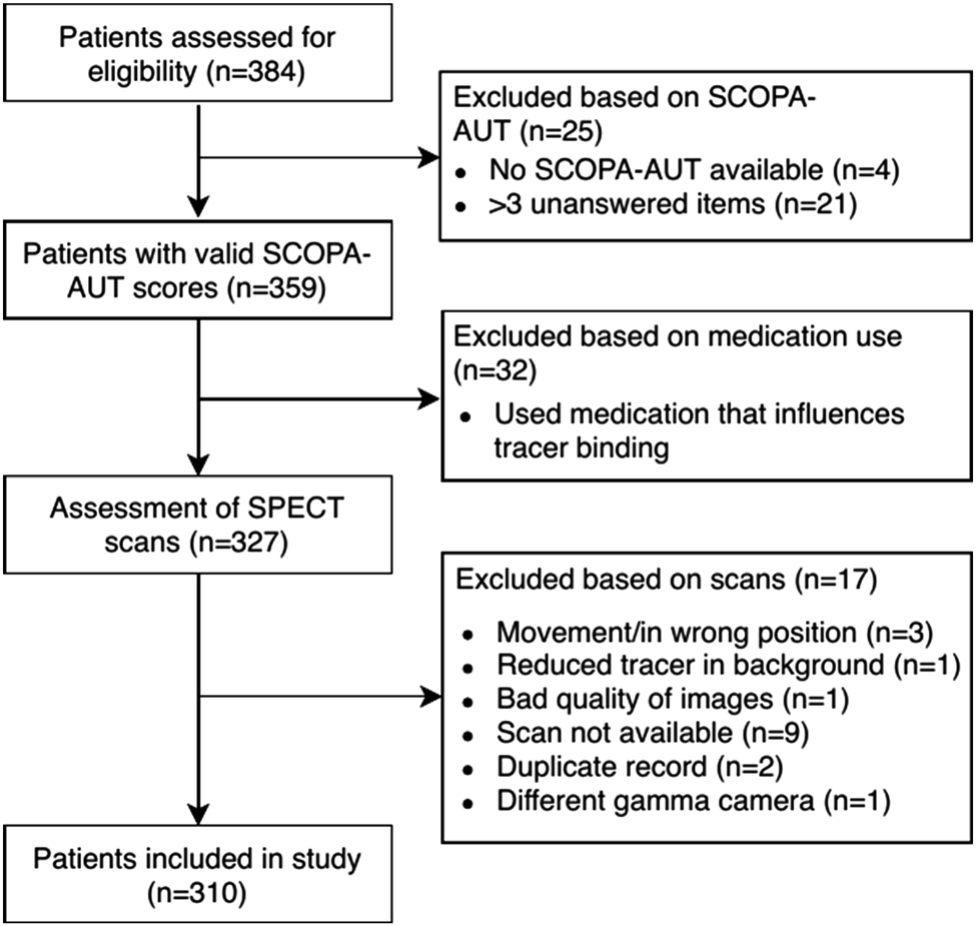
Inclusion and exclusion of patients. *SCOPA-AUT* Scales for Outcomes in Parkinson’s Disease—Autonomic, *SPECT* single-photon emission computed tomography

Patients were excluded if they were taking any medication during the [^123^I]FP-CIT SPECT scan that could influence the ability of the [^123^I]FP-CIT tracer to bind to DAT or SERT, such as SSRIs or serotonin–norepinephrine reuptake inhibitors (SNRIs) [28]. Patients without a valid SCOPA-AUT (Scales for Outcomes in Parkinson’s Disease—Autonomic) questionnaire to measure the severity of autonomic dysfunction were also excluded. Figure 1 shows the flowchart of inclusion and exclusion of patients.

### Measurements

Autonomic symptoms were evaluated using the SCOPA-AUT, a questionnaire designed and validated by Visser et al. [29] that evaluates the severity of gastrointestinal, urinary, cardiovascular, thermoregulatory, pupillomotor, and sexual symptoms. Only items 1–21 were used for these analyses, because items 22–25 regarding sexual dysfunction had low response rates. Up to three missing items were accepted, in which case the values were imputed with the mean score of the available items. We used the Unified Parkinson’s Disease Rating Scale—motor section (UPDRS-III) to assess the severity of motor symptoms of patients, thereby providing an indication of the disease severity [30]. The UPDRS-III was administered while patients were on dopaminergic medication. Imputation of missing data was not performed on the UPDRS-III, as this was considered to be unreliable for this scale. We also measured disease severity using the Hoehn and Yahr scale [31] and subjective disease duration. To quantify the medication usage of patients, the l-dopa equivalent daily dose (LEDD) was calculated [32]. To assess the global cognitive functioning of patients, we used the Mini-Mental State Examination (MMSE) [33]. These measurements were performed on the same day as the SPECT scan.

### Image acquisition and preprocessing

All patients orally received potassium perchlorate to prevent iodide uptake in the thyroid. A bolus of [^123^I]FP-CIT tracer was injected intravenously 3 h before the scan, with a dosage of approximately 185 MBq (specific activity > 185 MBq/nmol; radiochemical purity > 99%; produced as DaTSCAN according to GMP criteria at GE Healthcare, Eindhoven, The Netherlands). Static images were acquired using a single dual-head gamma camera with a fan-beam collimator, with a reconstructed voxel size of 3.9 mm^3^, and a pixel matrix of 128 × 128. After attenuation correction, filtered back projection with a Butterworth filter (order 8, cut-off 0.6 cycles/cm) was used for reconstruction of the scans. The scans were manually reoriented to align the left and right striatum according to the anterior–posterior commissure (AC-PC) line. Non-specific binding in the cerebellum was used as a reference (REF; WFU Pickatlas, AAL atlas; bilateral Crus II). Binding ratios were calculated, defined as the ratio between tracer bound specific to SERT or DAT and non-specific binding [(ROI − REF)/REF], to determine the availability of the SERT and DAT in extrastriatal and striatal regions, respectively.

### Regions of interest

The striatal left and right caudate nucleus and posterior putamen, the extrastriatal hypothalamus (left and right combined), midbrain, and left and right thalamus were defined as the regions of interest (ROIs). The caudate nucleus, midbrain, and thalamus were defined from the automated anatomical labelling (AAL) atlas. The posterior putamen ROI was based on the putamen ROI from the AAL atlas, in a way that it comprised only voxels posterior to the anterior commissure, as previously described [34]. This was done due to the existence of functional differences between the anterior and posterior region, and because the extent of dopamine depletion is more severe in the posterior putamen, correlating with the severity of motor symptoms [35]. The anterior putamen was not included in the analyses. The hypothalamus ROI was based on the Talairach Daemon (TD) Brodmann area + atlas, and was dilated twice due to its small size, as previously described [23]. All ROIs were implemented in the WFU Pickatlas (version 3.0.5; Wake Forest University).

### Data analysis

We used the Statistical Package for the Social Sciences (SPSS) version 22 (IBM Inc., Armonk, NY, USA) for the analyses. Assumptions for regression analyses were checked and met. Hierarchical multiple regression analyses with the ‘enter’ method were performed, with the SCOPA-AUT scores as the independent variable and [^123^I]FP-CIT binding ratios in each ROI as the outcome variables. Age was added as a covariate to the first step of the model to account for the decline in SERT and DAT availability with aging [36].

For the regression analyses, adjusted *p* values were calculated to correct for multiple comparisons with simple interactive statistician analysis (SISA, https://www.quantitativeskills.com/sisa/calculations/bonhlp.htm). This tool uses the mean association between variables that are mutually correlated (binding ratios in our ROIs) for the alpha correction (*r* = 0.73 for the extrastriatal ROIs, and *r* = 0.84 for the striatal ROIs), and allows for a less stringent correction than the Bonferroni method for multiple comparisons that assumes independence. For the four extrastriatal ROIs, this resulted in an adjusted statistical threshold of *p*_adj_ < 0.035 and for striatal ROIs this resulted in a threshold of *p*_adj_ < 0.040. A *p* value between *p*_adj_ and *p* = 0.050 was considered a trend.

Sensitivity analyses were done by separately adding measures of disease severity (UPDRS-III scores, H&Y stage or subjective disease duration), medication status or gender as covariates of no interest to the third step of the model. The analyses were also repeated in a subsample of unmedicated patients, and in patients with MMSE score > 24, because patients scoring equal to, or below 24 may suffer from cognitive impairments that impede their ability to correctly interpret or answer the questions of the SCOPA-AUT.

Post-hoc analyses were done with the cardiovascular, gastrointestinal, urinary, thermoregulatory, and pupillomotor subdomains of the SCOPA-AUT.

Statistical Parametric Mapping (SPM) version 12 imaging software was used to confirm the significant findings of the ROI analyses, using voxel-based analyses with age as a nuisance covariate. An explicit mask was placed for each ROI, in which we performed the voxel-based analysis. The masks were the same as in the ROI-based analysis. The statistical threshold was set to *p* < 0.05, family-wise error (FWE), corrected for multiple comparisons.

## Results

### Clinical characteristics

In total, 384 patients were assessed for eligibility. Due to a non-valid SCOPA-AUT, the use of SSRIs or SNRIs, or technical difficulties during scanning, 74 patients were excluded (see Fig. 1). The clinical characteristics of the remaining 310 patients that were included are summarized in Table 1.

**Table 1:** Sample characteristics

*N* patients (% male)	310 (62.6%)
Age (years)	66.35 (± 10.80)
SCOPA-AUT score	13.15 (± 7.84)
UPDRS-III score^a^	24.37 (± 12.52)
Hoehn and Yahr stage (in %)^b^	
0	1
1	15.2
1.5	6.8
2.0	40.6
2.5	18.1
3.0	6.8
4.0	2.9
5.0	1.0
Disease duration (years)^c^	3.68 (± 4.26)
MMSE score^d^	27.60 (± 3.24)
LEDD (mg/day)^e^	136.70 (± 258.09)
BDI score^f^	10.47 (± 7.59)
BR left thalamus	0.75 (± 0.18)
BR right thalamus	0.74 (± 0.19)
BR hypothalamus^g^	0.66 (± 0.21)
BR midbrain	0.76 (± 0.16)
BR left caudate nucleus	1.81 (± 0.43)
BR right caudate nucleus	1.86 (± 0.44)
BR left posterior putamen	1.59 (± 0.41)
BR right posterior putamen	1.48 (± 0.41)

For all variables except number of patients, the mean (standard deviation) is reported

*SCOPA-AUT* Scales for Outcomes in Parkinson’s Disease—Autonomic; *UPDRS-III* Unified Parkinson’s Disease Rating Scale, Sect. 3 (motor evaluation); *MMSE* Mini-Mental State Examination; *LEDD* levodopa equivalent daily dose; *BR* [^123^I]FP-CIT binding ratio

^a^24 patients had missing data

^b^24 patients had missing data

^c^63 patients had missing data

^d^40 patients had missing data

^e^12 patients had missing data

^f^10 patients had missing data

^g^1 patient had missing data

At least one autonomic symptom was experienced by 307 (99%) patients, of whom 260 (83.9%) had gastrointestinal symptoms, 304 (98.1%) had urinary symptoms, 171 (55.2%) had cardiovascular symptoms, 236 (76.12%) had thermoregulatory symptoms, and 122 (39.4%) had pupillomotor symptoms. The majority of patients (66.8%) were not yet using dopaminergic medication. SCOPA-AUT scores correlated positively with UPDRS-III scores (*r* = 0.316, *p* < 0.001), disease duration (*r* = 0.216, *p* < 0.001) age (*r* = 0.253, *p* < 0.001), and LEDD (*r* = 0.212, *p* < 0.001), likely reflecting increased autonomic dysfunctions with disease progression.

### ROI and voxel-based analyses

**Fig. 2:**
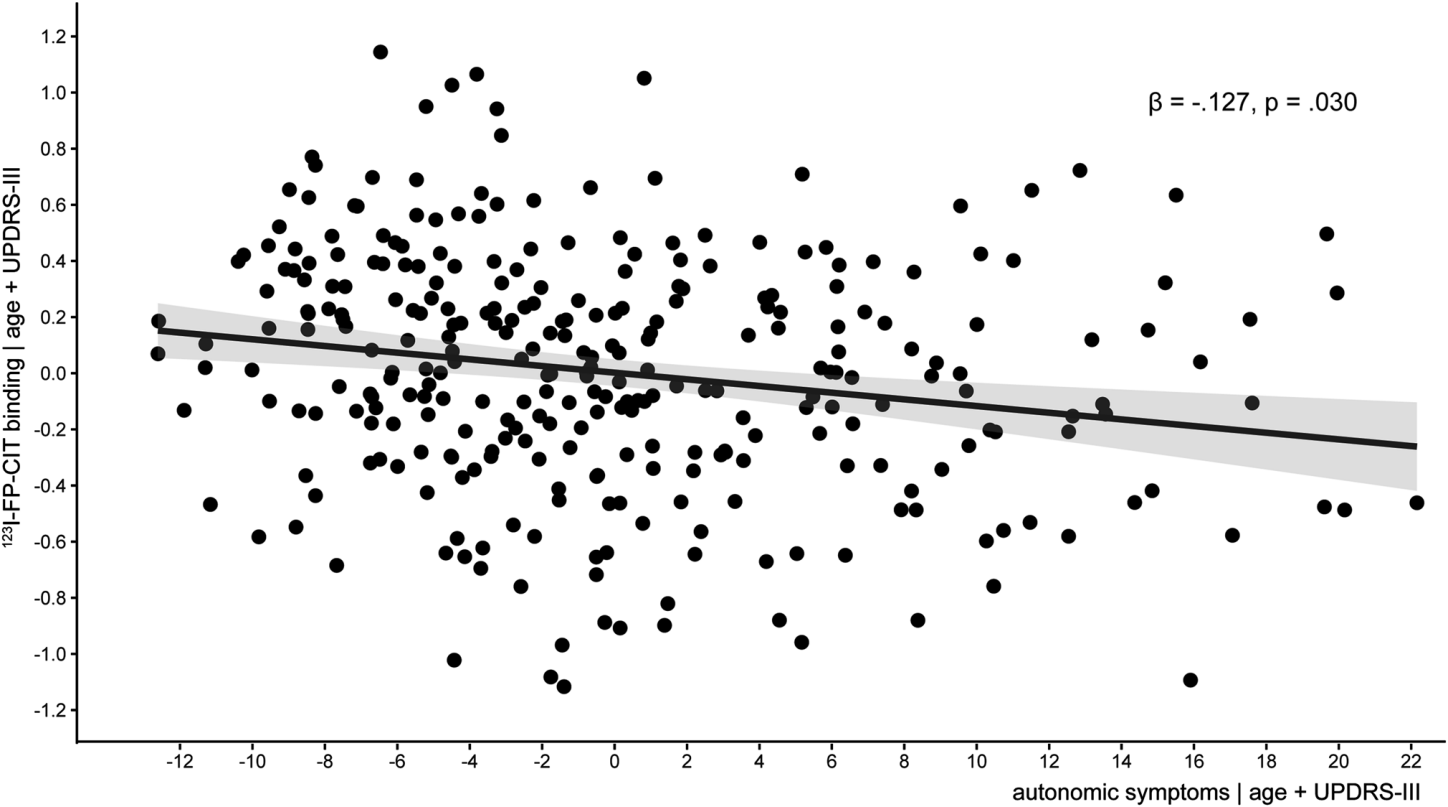
Partial plot of the association between [^123^I]FP-CIT binding ratios in the right caudate nucleus and SCOPA-AUT scores, corrected for age and UPDRS-III scores

In the total sample of PD patients, the SCOPA-AUT scores showed a significant negative association with [^123^I]FP-CIT binding ratios in the bilateral posterior putamen (left: *β* = − 0.143, *p* = 0.015, *R*^2 ^= 0.029, *ΔR*^2 ^= 0.019; right: *β* = − 0.147, *p* = 0.012, *R*^2 ^= 0.035, *ΔR*^2 ^= 0.020) and bilateral caudate nucleus (left: *β* = − 0.150 *p* = 0.006, *R*^2 ^= 0.140, *ΔR*^2 ^= 0.021; right: *β* = − 0.188, *p* = 0.001, *R*^2 ^= 0.102, *ΔR*^2 ^= 0.033). These associations all reflect a reduced striatal DAT availability with increasing autonomic dysfunction. A trend-significant negative association was found with SERT availability in the left thalamus and the hypothalamus (see Table 2). In line with these results, the SCOPA-AUT scores showed a significant negative association with voxel-based [^123^I]FP-CIT binding in the right caudate nucleus, particularly in the posterior caudate head (see supplementary Table 2 and Fig. 2). This relationship stayed intact when UPDRS-III was added to adjust for disease severity (*p*_FWE_ = 0.047, *T* = 3.45, *x*/*y*/*z* = 16/4/20). No significant association was observed for hypothalamic binding.

**Table 2:** Hierarchical multiple regression analyses with the SCOPA-AUT and each ROI

ROI	Step	Variable	*β*	*p*	*R*^2^	Δ*R*^2^
Left thalamus	1	Age	− 0.133	0.019	0.018	
	2	Age	− 0.102	0.081		
		SA	**− 0.122**	**0.036**	**0.032**	**0.014**
Right thalamus	1	Age	− 0.169	0.003	0.029	
	2	Age	− 0.162	0.006		
		SA	− 0.029	0.624	0.029	0.001
Hypothalamus	1	Age	− 0.212	0.000	0.045	
	2	Age	− 0.183	0.002		
		SA	**− 0.116**	**0.045**	**0.057**	**0.013**
Midbrain	1	Age	− 0.041	0.469	0.002	
	2	Age	− 0.025	0.671		
		SA	− 0.064	0.276	0.006	0.004
Left caudate nucleus	1	Age	− 0.345	0.000	0.119	
	2	Age	− 0.307	0.000		
		SA	**− 0.150**	**0.006**	**0.140**	**0.021**
Right caudate nucleus	1	Age	− 0.263	0.000	0.069	
	2	Age	− 0.215	0.000		
		SA	**− 0.188**	**0.001**	**0.102**	**0.033**
Left posterior putamen	1	Age	− 0.102	0.072	0.010	
	2	Age	− 0.066	0.256		
		SA	**− 0.143**	**0.015**	**0.029**	**0.019**
Right posterior putamen	1	Age	− 0.122	0.032	0.015	
	2	Age	− 0.084	0.147		
		SA	**− 0.147**	**0.012**	**0.035**	**0.020**

Hierarchical model, method: enter. *N* = 310 for all except hypothalamus, where *N* = 309. SA = SCOPA-AUT score, *β* = standardized regression coefficient, *p* = significance, *R*^2^ = proportion of variance of independent variable explained by the regression model. Δ*R*^2^ = difference between *R*^2^ of model 1 and model 2. The most relevant results are highlighted in bold

### Sensitivity analyses

When separately adding the UPDRS-III score (*N* = 287, see Fig. 2) and medication status (*N* = 298) to the model, only the associations between autonomic dysfunction and [^123^I]FP-CIT binding ratios in the right caudate nucleus remained (UPDRS-III: *β* = − 0.127, *p* = 0.030, *R*^2 ^= 0.174, *ΔR*^2 ^= 0.067; medication status: *β* = − 0.131, *p* = 0.025, *R*^2 ^= 0.131, *ΔR*^2 ^= 0.029). Using Hoehn and Yahr stage or disease duration to correct for disease severity instead of the UPDRS-III score did not change these results (Hoehn and Yahr: *β* = − 0.156, *p* = 0.010, *R*^2 ^= 0.160, *ΔR*^2 ^= 0.035; disease duration: *β* = − 0.147, *p* = 0.021, *R*^2 ^= 0.161, *ΔR*^2 ^= 0.048). Adding gender as a covariate also did not significantly alter the results.

Additional analyses were done with subsamples of patients. When patients with signs of dementia (MMSE ≤ 24; *N* = 64) were excluded, SCOPA-AUT scores showed a statistically significant negative association with [^123^I]FP-CIT binding ratios in the left thalamus (*β* = − 0.169, *p* = 0.011, *R*^2 ^= 0.040, *ΔR*^2 ^= 0.026), left posterior putamen (*β* = − 0.152, *p* = 0.022, *R*^2 ^= 0.024, *ΔR*^2 ^= 0.021), and right caudate nucleus (*β* = − 0.171, *p* = 0.008, *R*^2 ^= 0.088, *ΔR*^2 ^= 0.027). SCOPA-AUT scores no longer showed a significant association with [^123^I]FP-CIT binding ratios when only medication-free PD patients were considered (*N* = 207).

### Subdomains of autonomic dysfunction

To determine which autonomic symptoms drove the associations, post hoc analyses were performed with subdomains of the SCOPA-AUT. These results showed that the reported associations with the total SCOPA-AUT (excluding sexual dysfunctions) were mainly driven by cardiovascular and gastrointestinal symptoms (see supplementary Table 1). Also here, adding UPDRS-III scores to adjust for the influence of disease severity, only [^123^I]FP-CIT binding in the right caudate nucleus was significantly associated with autonomic symptoms (cardiovascular symptoms: *β* = − 0.124, *p* = 0.027, *R*^2 ^= 0.175, *ΔR*^2 ^= 0.074; gastrointestinal symptoms: *β* = − 0.187, *p* = 0.001, *R*^2 ^= 0.190, *ΔR*^2 ^= 0.053; see supplementary Fig. 1).

### Post hoc analyses

One of our previous studies in an overlapping sample showed that DAT availability in the right caudate nucleus was also negatively associated with depressive symptoms [37]. Because of potential spatial overlap with the current results, additional post hoc analyses were performed to check for the influence of depression, using the Beck Depression Inventory (BDI). When BDI scores were added to the third step of the model, [^123^I]FP-CIT binding in the right caudate nucleus was no longer associated with SCOPA-AUT scores. Nevertheless, our voxel-based analysis (see supplementary results) showed that the BDI scores were associated with a different—more anteroventral—region within the caudate nucleus (*x*/*y*/*z*: 16/16/− 6), than the SCOPA-AUT (*x*/*y*/*z*: 16/4/20; see supplementary Figs. 2 and 3).

## Discussion

The aim of this study was to elucidate the association between extrastriatal serotonergic and striatal dopaminergic degeneration and the severity of autonomic symptoms. Our results confirm our hypothesis that reduced DAT availability in the striatum, particularly the caudate nucleus, is associated with more autonomic symptoms in PD. This association was corroborated by our voxel-based analysis and seems mainly driven by cardiovascular and gastrointestinal symptoms. We also hypothesized that reduced extrastriatal SERT availability would be associated with increased autonomic failure. However, a weaker relationship was found here, with only a trend-significant relationship in the left thalamus and hypothalamus. When correcting for disease severity, only the relationship in the right caudate nucleus remained intact.

Previous research has suggested the involvement of striatal dopaminergic neurons in a wide range of autonomic dysfunctions, including urinary dysfunction [6, 38] and hyposalivation [19]. Our association with gastrointestinal symptoms is in accordance with a previous study that suggested the involvement of striatal D2 receptors in the salivary response through signalling via the globus pallidus and mesencephalic reticular formation to the sympathetic preganglionic neurons in the spinal cord [19]. A recent DaT study also found that gastrointestinal dysfunction, especially constipation, is associated with striatal dopaminergic degeneration [26]. In accordance with our findings, their association was stronger in the caudate nucleus than in the putamen. Our cardiovascular findings are in accordance with a previous study describing the involvement of striatal dopamine receptors in central regulation of blood pressure and heart rate, possibly through parasympathetic nervous activity [21]. Nevertheless, our results conflict with those found by Goldstein et al. [39], who reported that the occurrence of cardiac denervation is not related to the severity of striatal dopaminergic degeneration in PD patients (*N* = 77). Another previous DAT study investigating the association between autonomic dysfunction and striatal dopamine depletion found that urinary symptoms, but no other autonomic symptoms, were associated with reduced dopaminergic activity in the putamen [27].

When the association between [^123^I]FP-CIT binding ratios and autonomic symptoms was adjusted for disease severity, only the right caudate nucleus showed a relationship with autonomic dysfunction. It should be noted that, as Braak et al. [40] proposed, autonomic symptoms often begin before the onset of motor symptoms, and, therefore, using motor symptoms to correct for disease severity may not be entirely accurate. Most patients in this sample were in similar disease stages (H&Y stage 2) when the SPECT images were acquired, so adjusting for UPDRS-III scores may have resulted in over-correction for disease severity. However, when using Hoehn and Yahr stage or subjective disease duration to correct for disease severity, the results remained the same. When the analyses were repeated with only unmedicated patients, no relationships remained significant, but this may be due to the reduction in power.

Even though our extrastriatal findings were less robust and did not survive the adjustment for disease severity, they do support previous research suggesting the involvement of serotonergic degeneration in autonomic dysfunction. A previous study from our group suggested that loss of SERT-expressing neurons in the hypothalamus may account for dysregulation of the autonomic nervous system in other parkinsonian disorders such as multiple system atrophy with Parkinsonian features (MSA-P) and progressive supranuclear palsy (PSP) [23]. Previous studies also described a relationship between blood pressure regulation and serotonin levels in the dorsomedial hypothalamus and nucleus tractus solitarius, which has projections to the hypothalamus, thalamus, and midbrain [14, 41].

This study was one of the first to investigate the association between dopaminergic and serotonergic degeneration in (extra)striatal areas, especially the hypothalamus, and autonomic symptoms in PD using [^123^I]FP-CIT SPECT. A strength is the large sample size of 310 patients. Another strength is the exclusion of patients taking SSRIs or SNIRs, as these increase binding of the tracer to DAT by about 10% [28]. An additional strength is the corroboration of our findings using voxel-based techniques.

A possible limitation is the use of the SCOPA-AUT, which is a self-report questionnaire. Healthy (elderly) controls are also known to score points on the SCOPA-AUT, suggesting that not all autonomic dysfunction measured is necessarily due to the PD pathology [29]. Nevertheless, it has been validated independently and was found to be an acceptable, precise, and consistent scale to assess autonomic symptoms in PD [42]. A second limitation is the use of a single radiotracer to study both striatal dopaminergic and extrastriatal serotonergic binding (although this does reduce the radiation load).

More research on the mechanisms behind autonomic dysfunction in PD is needed to confirm our findings. Our study provides a first look into the associations between dopaminergic and serotonergic degeneration and autonomic dysfunction, but the observed relationships are modest and require replication. Additionally, further research is needed to investigate the specific subregion within the caudate nucleus that is associated with autonomic symptoms seen in PD, as our post hoc voxel-based analyses show that the severity of depressive and autonomic symptoms is associated with distinct parts of the caudate nucleus. Our results are also in need of replication with a tracer that is selective to SERT, such as PET scans using [^11^C]-3-amino-4-(2-dimethylaminomethylphenylsulfanyl)-benzonitrile, or [^11^C]DASB [43], to draw clearer conclusions about the specific involvement of serotonergic degeneration in autonomic dysfunction.

In conclusion, autonomic symptoms in PD, particularly cardiovascular and gastrointestinal dysfunction, were associated with lower striatal [^123^I]FP-CIT binding, mainly in the right caudate nucleus. These may give a first indication that degeneration of the dopaminergic projection towards the caudate nucleus is modestly involved in autonomic dysfunction in PD patients, although the direction of causality and the underlying mechanisms need further study.

## Electronic supplementary material

Below is the link to the electronic supplementary material.

Supplementary file1 (DOC 457 kb)
