# Supplementary material for: Autonomic failure in Parkinson’s disease is associated with striatal dopamine deficiencies
Source: J Neurol. 2020 Mar 12;267(7):1922–30. doi: 10.1007/s00415-020-09785-5 (PMC7320937; doi:10.1007/s00415-020-09785-5)
Supplement: Supplementary file 1 — Supplementary file1 (DOC 457 kb) [file 415_2020_9785_MOESM1_ESM.doc]

**Supplementary online content**

Content

Supplementary Table1 - *Hierarchical multiple regression analyses with SCOPA-AUT subdomains and each ROI*

Supplementary Table2 - *Voxel-based analyses for SCOPA-AUT scores and the right caudate nucleus*

Supplementary Table3 - *Voxel-based analyses for BDI scores and the right caudate nucleus*

Supplementary Figure1 - *Associations between the right caudate nucleus and SCOPA-AUT subdomains*

Supplementary Figure2 - *Voxel-based analyses for SCOPA-AUT and BDI scores, and the right caudate nucleus*

Supplementary Results - *Post-hoc analysis: BDI scores and binding in the right caudate nucleus.*

| **Supplementary Table1 - Hierarchical multiple regression analyses with SCOPA-AUT subdomains and each ROI** | | | | | | | | | | | | | | | | | | | |
| --- | --- | --- | --- | --- | --- | --- | --- | --- | --- | --- | --- | --- | --- | --- | --- | --- | --- | --- | --- |
|  |  |  | Cardiovascular | | | Gastrointestinal | | | Thermoregulatory | | | | Urinary | | | | Pupillomotor | | |
| **ROI** | Step | Variable | β | *p* | R^2^ | β | *p* | R^2^ | β | *p* | R^2^ | β | | *p* | R^2^ | β | | *p* | R^2^ |
| **Left thalamus** | 1 | Age | -.133 | .019 | .018 | -.133 | .019 | .018 | -.133 | .019 | .018 | -.133 | | .019 | .018 | -.133 | | .019 | .018 |
|  | 2 | Age | -.117 | .038 |  | -.107 | .062 |  | -.136 | .017 |  | -.122 | | .043 |  | -.115 | | .042 |  |
|  |  | **SA** | **-.132** | **.020** | **.035** | **-.145** | **.011** | **.038** | -.043 | .448 | .019 | -.032 | | .599 | .018 | **-.140** | | **.013** | **.037** |
| **Right thalamus** | 1 | Age | -.169 | .003 | .029 | -.169 | .003 | .029 | -.169 | .003 | .029 | -.169 | | .003 | .029 | -.169 | | .003 | .029 |
|  | 2 | Age | -.160 | .005 |  | -.157 | .006 |  | -.168 | .003 |  | -.180 | | .003 |  | -.163 | | .004 |  |
|  |  | SA | -.086 | .129 | .036 | -.070 | .222 | .033 | -.017 | .766 | .029 | -.030 | | .612 | .029 | -.054 | | .340 | .032 |
| **Hypothalamus** | 1 | Age | -.212 | .000 | .045 | -.212 | .000 | .045 | -.212 | .000 | .045 | -.212 | | .000 | .045 | -.212 | | .000 | .045 |
|  | 2 | Age | -.198 | .000 |  | -.192 | .001 |  | -.212 | .000 |  | -.181 | | .002 |  | -.207 | | .000 |  |
|  |  | **SA** | **-.123** | **.028** | **.060** | -.111 | .051 | .057 | -.003 | .958 | .045 | -.093 | | .119 | .053 | -.041 | | .465 | .047 |
| **Midbrain** | 1 | Age | -.041 | .469 | .002 | -.041 | .469 | .002 | -.041 | .469 | .002 | -.041 | | .469 | .002 | -.141 | | .469 | .002 |
|  | 2 | Age | -.028 | .619 |  | -.026 | .652 |  | -.042 | .461 |  | -.047 | | .443 |  | -.024 | | .678 |  |
|  |  | **SA** | **-.113** | **.049** | **.014** | -.085 | .144 | .009 | -.012 | .838 | .002 | -.016 | | .796 | .002 | **-.143** | | **.012** | **.022** |
| **Left caudate nucleus** | 1 | Age | -.345 | .000 | .119 | -.345 | .000 | .119 | -.345 | .000 | .119 | -.345 | | .000 | .119 | -.345 | | .000 | .119 |
|  | 2 | Age | -.325 | .000 |  | -.305 | .000 |  | -.343 | .000 |  | -.319 | | .000 |  | -.341 | | .000 |  |
|  |  | **SA** | **-.169** | **.002** | **.147** | **-.221** | **.000** | **.166** | -.020 | .710 | .119 | -.076 | | .180 | .124 | -.030 | | .579 | .120 |
| **Right caudate nucleus** | 1 | Age | -.263 | .000 | .069 | -.263 | .000 | .069 | -.263 | .000 | .069 | -.263 | | .000 | .069 | -.263 | | .000 | .069 |
|  | 2 | Age | -.242 | .000 |  | -.220 | .000 |  | -.265 | .000 |  | -.228 | | .000 |  | -.255 | | .000 |  |
|  |  | **SA** | **-.183** | **.001** | **.102** | **-.239** | **.000** | **.124** | -.019 | .727 | .070 | -.104 | | .076 | .079 | -.069 | | .215 | .074 |
| **Left posterior putamen** | 1 | Age | -.102 | .072 | .010 | -.102 | .072 | .010 | -.102 | .072 | .010 | -.102 | | .072 | .010 | -.102 | | .072 | .010 |
|  | 2 | Age | -.085 | .133 |  | -.064 | .260 |  | -.103 | .073 |  | -.081 | | .178 |  | -.100 | | .081 |  |
|  |  | **SA** | **-.150** | **.009** | **.033** | **-.215** | **.000** | **.055** | -.004 | .941 | .010 | -.062 | | .301 | .014 | -.016 | | .774 | .011 |
| **Right posterior putamen** | 1 | Age | -.122 | .032 | .015 | -.122 | .032 | .015 | -.122 | .032 | .015 | -.122 | | .032 | .015 | -.122 | | .032 | .015 |
|  | 2 | Age | -.105 | .064 |  | -.084 | .135 |  | -.121 | .033 |  | -.097 | | .108 |  | -.115 | | .045 |  |
|  |  | **SA** | **-.145** | **.011** | **.035** | **-.207** | **.000** | **.056** | -.001 | .980 | .015 | -.074 | | .219 | .020 | -.055 | | .337 | .018 |

*Note.* Hierarchical model, method: Enter. N = 310 for all except hypothalamus, where N = 309. SA = SCOPA-AUT scores, *β* = standardized regression coefficient, *p* = significance, R^2^ = proportion of variance of independent variable explained by the regression model. The significant results are made bold.

| **Supplementary Table 2 - Voxel-based analyses for SCOPA-AUT scores and the right caudate nucleus** | | | | |
| --- | --- | --- | --- | --- |
| **Region** | K_e_ | *P*_FWE_ peak-voxel | *T* | *x/y/z (mm)* |
| **Right caudate nucleus**  **(df, 1,307)** | 17 | <.001 | 4.92 | 16/4/20 |
|  | 28 | .001 | 4.48 | 10/10/12 |
|  | 4 | .004 | 4.12 | 8/10/4 |
|  | 2 | .009 | 3.91 | 20/18/8 |
|  | 3 | .038 | 3.47 | 18/18/2 |
|  | 3 | .046 | 3.41 | 20/14/14 |
|  | 2 | .049 | 3.38 | 12/10/-8 |

*Note.* df = degrees of freedom; K_e_ = cluster extent in number of voxels; *P*_FWE_ = familywise error-corrected *P* values; *T* = T statistic; x/y/z = location of peak voxel of cluster.

| **Supplementary Table 3 - Voxel-based multiple regression for BDI scores and the right caudate nucleus** | | | | |
| --- | --- | --- | --- | --- |
| **Region** | K_e_ | *P*_FWE_ peak-voxel | *T* | *x/y/z (mm)* |
| **Right caudate nucleus**  **(df, 1,297)** | 118 | <.001 | 5.38 | 16/16/-6 |
|  | 3 | .010 | 5.24 | 10/12/-12 |
|  | 3 | .013 | 4.21 | 14/2/18 |

*Note*. df = degrees of freedom; Ke = cluster extent in number of voxels; P_FWE_ = familywise error-corrected P values; T = T statistic; x/y/z = location of peak voxel of cluster.

**Supplementary Figure 1**


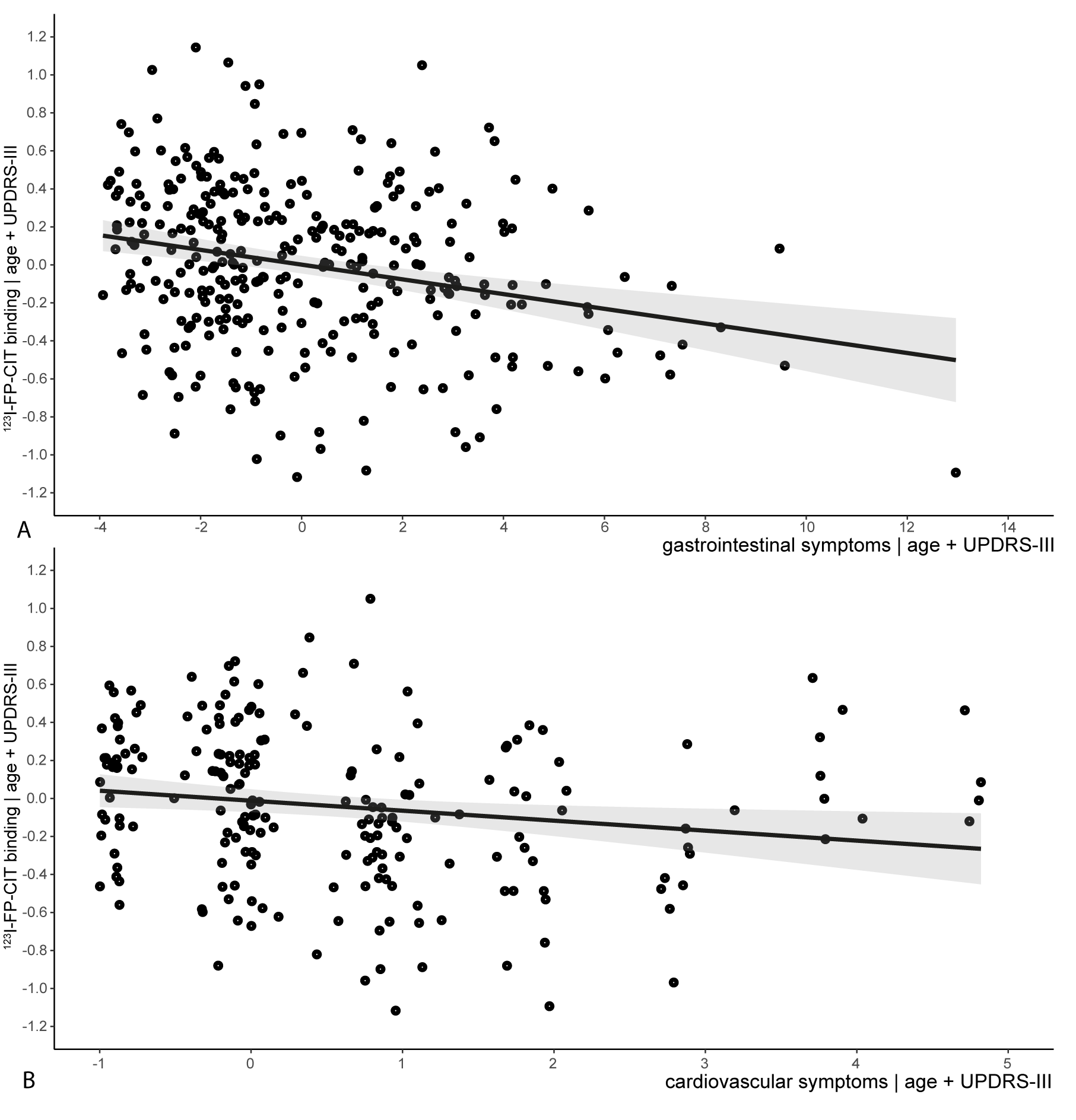
S**upplementary Figure 1**: Partial plots of the association between [^123^I]FP-CIT binding in the right caudate nucleus and gastrointestinal symptoms (above) and cardiovascular symptoms (below), corrected for age and UPDRS-III scores.


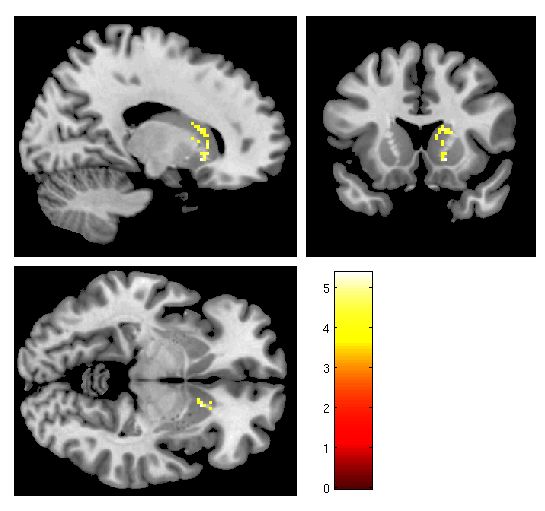

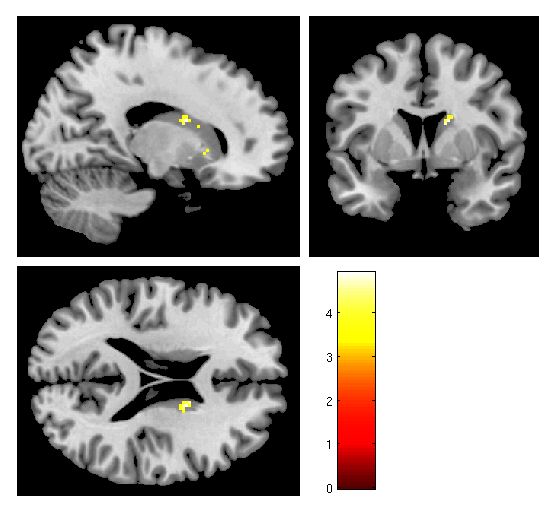
Supplementary Figure 2

**Supplementary Figure 2**: Voxel-by-voxel analysis showing the negative association between SCOPA-AUT (left) and BDI (right) scores and [^123^I]FP-CIT binding ratios in the right caudate nucleus at (x,y,z), 16,4,20 and 16,16,-6, respectively.

**Supplementary Results**

A post-hoc analysis was done to examine the relationship between the BDI scores and the [^123^I]FP-CIT binding ratios in the right caudate nucleus. A voxel-based regression analysis was performed in SPM with the BDI scores as the independent variable, and age as a covariate. Increased depressive symptoms as measured by the BDI showed a significant negative relationship with [^123^I]FP-CIT binding ratios (see supplementary Table3 and supplementary Figure3).
